# Multimodal MRI-based classification of migraine: using deep learning convolutional neural network

**DOI:** 10.1186/s12938-018-0587-0

**Published:** 2018-10-11

**Authors:** Hao Yang, Junran Zhang, Qihong Liu, Yi Wang

**Affiliations:** Department of Medical Information Engineering, School of Electrical Engineering and Information, Sichuan University, Chengdu, Sichuan China

**Keywords:** Deep learning, Migraine, Diagnosis, Resting-state functional MRI, Convolutional neural networks

## Abstract

**Background:**

Recently, deep learning technologies have rapidly expanded into medical image analysis, including both disease detection and classification. As far as we know, migraine is a disabling and common neurological disorder, typically characterized by unilateral, throbbing and pulsating headaches. Unfortunately, a large number of migraineurs do not receive the accurate diagnosis when using traditional diagnostic criteria based on the guidelines of the International Headache Society. As such, there is substantial interest in developing automated methods to assist in the diagnosis of migraine.

**Methods:**

To the best of our knowledge, no studies have evaluated the potential of deep learning technologies in assisting with the classification of migraine patients. Here, we used deep learning methods in combination with three functional measures (the amplitude of low-frequency fluctuations, regional homogeneity and regional functional correlation strength) based on rs-fMRI data to distinguish not only between migraineurs and healthy controls, but also between the two subtypes of migraine. We employed 21 migraine patients without aura, 15 migraineurs with aura, and 28 healthy controls.

**Results:**

Compared with the traditional support vector machine classifier, which has an accuracy of 83.67%, our Inception module-based convolutional neural network approach showed a significant improvement in classification output (over 86.18%). Our data also indicate that the Inception module-based CNN performs better than the AlexNet-based CNN (Inception module-based CNN reached an accuracy of 99.25%). Finally, we also found that regional functional correlation strength (RFCS) could be regarded as the optimum input out of the three indices (ALFF, ReHo, RFCS).

**Conclusions:**

Overall, our study shows that combining the three functional measures of rs-fMRI with deep learning classification is a powerful method to distinguish between migraineurs and healthy individuals. Our data also highlight that deep learning-based frameworks could be used to develop more complicated models or systems to aid in clinical decision making in the future.

**Electronic supplementary material:**

The online version of this article (10.1186/s12938-018-0587-0) contains supplementary material, which is available to authorized users.

## Background

A migraine is a common, chronic, incapacitating neurovascular disorder that is characterized by attacks of severe headaches, autonomic nervous system dysfunction, and in some patients, aura-associated neurologic symptoms [1]. A recent survey by the World Health Organization highlighted that migraine ranks as the third most prevalent disorder, affecting roughly 12% of adults globally. Moreover, people who experience migraines may have an increased risk of ischemic stroke, unstable angina, and/or affective disorder [2–5]. In migraine without aura (MWoA), previously known as common migraine, attacks are usually associated with nausea, vomiting, and/or sensitivity to light, sound, or movement. The other major subtype of migraine, which is known as migraine with aura (MWA), is accompanied by early symptoms. As the diagnosis of migraines is based on a combination of features and it is relatively difficult to exclude possible causes, achieving an accurate diagnosis using traditional methods (e.g., symptoms analysis, medical tests) is not easy. In fact, according to American Migraine Studies, only 65.2% of migraineurs receive the correct diagnosis [6]. Undoubtedly, there has been substantial interest in developing automated methods with the potential to assist in the diagnosis of migraines.

In recent years, resting-state functional magnetic resonance imaging (rs-fMRI) has attracted considerable attention for studying neural mechanisms. This approach not only overcomes the potential limitation associated with task paradigms in fMRI studies, but is also a non-invasive imaging technique capable of measuring spontaneous brain activity. In this approach low-frequency fluctuations in blood oxygen level-dependent (BOLD) signals are used to identify areas of increased or decreased neuronal activity [7–9]. By using resting-state fMRI, researchers have demonstrated that migraines are related to different indices of functional brain alterations, including amplitude of low-frequency fluctuations (ALFF), regional homogeneity (ReHo), and regional functional correlation strength (RFCS). For instance, compared with the healthy subjects, migraineurs showed significant changes in ALFF within the anterior cingulate cortex and prefrontal cortex, Moreover, a correlation analysis demonstrated that in migraineurs, ReHo values changes in the prefrontal cortex [10], and orbitofrontal cortex [11]. Despite these results demonstrating that migraines might contribute to functional brain alterations due to the repetitive occurrence of pain-related processes, very few studies have considered the possibility of using these functional features to improve the classification and diagnosis of migraines.

Deep learning is a relatively new technique in the field of computational medical imaging [12, 13]. This technique automatically creates multilevel models with hierarchical representations of the input data and permits powerful automatic feature extraction. Deep learning models are being used more frequently to improve diagnostic abilities and to discover the diverse patterns in patients’ data that are characteristic of a disease [14, 15]. Convolutional neural networks (CNNs) is a deep learning model that has been applied successfully in the diagnosis of diseases based on MRI. For instance, Li et al. [16] constructed a CNN with two convolutional layers and one fully connected layer to identify the Alzheimer’s disease (AD). They obtained a final recognition rate of up to 92.87% when comparing AD and healthy controls (HC). Similarly, Sarraf et al. [17] were able to make early diagnosis of Alzheimer’s disease based on GoogleNet [18], a successful network that is broadly used for object recognition and classification. This network based on a modern design module called “Inception”, and this module was come up with to enhance the performance of classifier, which resulted in a higher accurate, reaching to 98.74% between AD and HC [19]. These deep learning-based frameworks have implications for numerous applications in classifying brain disorders in clinical trials and large-scale research studies. In light of these previous findings, it is evident that the classification accuracy for a specific disease can be further improved by choosing the proper deep learning model.

Here, we used two deep learning-based classifiers to distinguish between healthy brains, brains affected by MWA and brains affected by MWoA. The first model we used was the CNN network based on AlexNet [21], and the second model was the CNN with Google’s Inception module. As a lot of information can be lost when decomposing 4D rs-fMRI data into 2D data, many fMRI studies use feature mapping instead of raw data as the original input [17, 20]. Thus, after preprocessing, we extracted three indices—ALFF, ReHo and RFCS—as inputs to improve the classification results for migraine patients. We compare the classification results obtained using a 3-way classifier (MWA vs. MWoA vs. HC) and two binary classifiers (migraines vs. HC, and MWA vs. MWoA). To the best of our knowledge, this is one of the first studies to examine the performance of different deep learning-based frameworks and to apply them specifically for migraine discrimination. Taken together, our results could help improve diagnostic accuracy for patients suffering from migraines.

## Methods

### Subjects

Our study population included 21 migraine patients without aura, 15 migraineurs with aura, and 28 healthy controls. The age and gender differences between the three groups were tested using the t-test and no significant difference was observed (p > 0.05). In addition, the diagnosis of migraineurs was made by neurologic practitioners according to the criteria from the Second Edition of the International Classification of Headache Disorders (ICHD-II) [22]. All subjects were right-handed, aged between 18 and 50 years, and underwent brain scans at the Huaxi MR Research Center of the West China Hospital. The patients were attack-free for at least 72 h prior to the brain scan, and 48 h after the scan. Patients with chronic migraine, other chronic or current pain disorders, a history of mental disease, or other neurological disorders were excluded from this study. The study was approved by the Medical Ethics Committee of the West China Hospital, Sichuan University, and written informed consent was obtained from each participant. Table 1 lists the demographic and clinical data of the 64 subjects.

**Table 1:** Demographic and clinical characteristics of the 64 participants

Variables (mean ± SD)	MWoA	MWA	HC	p-value		
				MWoA vs. HC	MWA vs. HC	MWoA vs. MWA
Sex (male/female)	7/14	4/11	13/15	0.366	0.216	0.679
Age (years)	29.67 ± 6.45	32.2 ± 7.68	31.57 ± 6.72	0.323	0.782	0.291
Education (years)	16.90 ± 4.40	15.07 ± 1.98	16.36 ± 2.87	0.601	0.129	0.141
24-HAMD	5.95 ± 6.76	8.73 ± 5.13	3.57 ± 2.41	0.012	0.002	0.190
14-HAMA	4.28 ± 5.53	7.47 ± 6.13	2.07 ± 2.38	0.031	0.005	0.113

*SD* standard deviation, *HAMD* Hamilton Depression Scale, *HAMA* Hamilton Anxiety Scale

### Data preprocessing

All data were acquired using a 3.0 Tesla MRI system (Trio Tim, Siemens, Erlangen, Germany). Subjects were instructed simply to rest with their eyes closed, not to think of anything, and not to fall asleep. Imaging data were collected transversely by using an echo-planar imaging (EPI) sequence with the following settings: repetition time/echo time (TR/TE) = 1900/2.26 ms, flip angle = 9°, slice thickness/gap = 1/0 mm, field of view (FOV) = 256 × 256 mm^2^, matrix = 256 × 256, and voxel size = 1 × 1×1 mm^3^. The rs-fMRI data were also collected using an echo planar imaging (EPI) sequence but with the following settings: TR/TE = 2000/30 ms, flip angle = 90°, slice thickness/gap = 5/0 mm, FOV = 240 × 240 mm^2^, matrix = 64 × 64, and voxel size = 3.75 × 3.75 × 5 mm^3^. Resting-state functional images were analyzed with the Statistical Parametric Mapping software (SPM8, http://www.fil.ion.ucl.ac.uk/spm) and the Data Processing Assistant for Resting-State fMRI (DPARSF, http://rfmri.org/DPARSF) toolbox. The first 10 EPI volumes of each functional data were discarded due to instability of the initial MRI signal and adaptation of the participants to the situation. The remaining volumes first underwent slices-timing correction, and were then realigned to the first volume to correct for susceptibility-by-movement interaction. None of the subjects’ heads in this study had a movement in any direction that exceeded a 2-mm displacement or a 2° of rotation. Then, the resulting images were spatially normalized into a standard stereotaxic space at 3-mm isotropic voxels using the Montreal Neurological Institute (MNI) template. Next, band-pass-filtering (0.01–0.08 Hz) was performed on the time series of each voxel to reduce the effect of low-frequency drifts and high-frequency physiological noise [23]. Finally, the ALFF, ReHo and RFCS were calculated as described below.

### Feature mapping

ALFF is an effective indicator of regional intrinsic or spontaneous neuronal activity in the brain [24]. The time series for each voxel was first transformed to the frequency domain using a Fast Fourier Transform, and the power spectrum was obtained. The square root of the power spectrum was computed, and subsequently averaged across a predefined frequency interval. When calculating ALFF, the normalized and resliced images were smoothed using a 4-mm FWHM Gaussian kernel. In our study, the ALFF within the 0.01–0.08 Hz frequency band was calculated for each voxel using the Resting-State fMRI Data Analysis Toolkit (REST, http://rest.restfmri.net). To reduce the global effects of variability across participants, the ALFF of each voxel for a certain subject was divided by the global mean ALFF value. ReHo was used to measure the similarity between the time series of a given voxel and that of its nearest neighbor. It is calculated using Kendall’s coefficient of concordance, which produces reliable results in rs-fMRI data analysis. In our study, we used cubic cluster of 27 voxels for each normalized and resliced image and assigned the ReHo value of every cubic cluster to the central voxel. A larger ReHo value for a given voxel indicates a higher local synchronization of rs-fMRI signals among neighboring voxels. As for ALFF, the ReHo of each voxel was also divided by the global mean ReHo value for each subject. All these procedures were performed using REST software.

By measuring functional connectivity (FC) of the distinct brain regions, rs-fMRI can be used to evaluate the brain function. To reduce the effect of the region of interest (ROI) selection strategy on the FC results, we employed the RFCS method, which measures the extent of the average correlation of a given brain region with all other regions [25]. To compute resting-state FC, we repressed the spurious effects of nuisance covariates [26]. We parcellated the rs-fMRI into 116 ROIs according to the Automated Anatomical Labeling (AAL) template, and thereby obtained 116 FC results for each subject. The RFCS values were then calculated using a previously described method [27]. The RFCS was defined as:

1 $$S\text{(}i\text{) = }\frac{\text{1}}{N}\sum\limits_{j} {\left| {R_{j} } \right|}$$

where, *R*_*j*_ is the result of the FC in a certain region of the AAL, *S*_*i*_ represents the average activation level, and the N is the number of regions.

For each subject, we therefore obtained three functional maps: ALFF, ReHo and RFCS. To some extents, three features respectively reflect the degree of activity, the degree of synchronization and the degree of global synchronization of spontaneous neuronal activity.

### Convolutional neural network

**Fig. 1:**
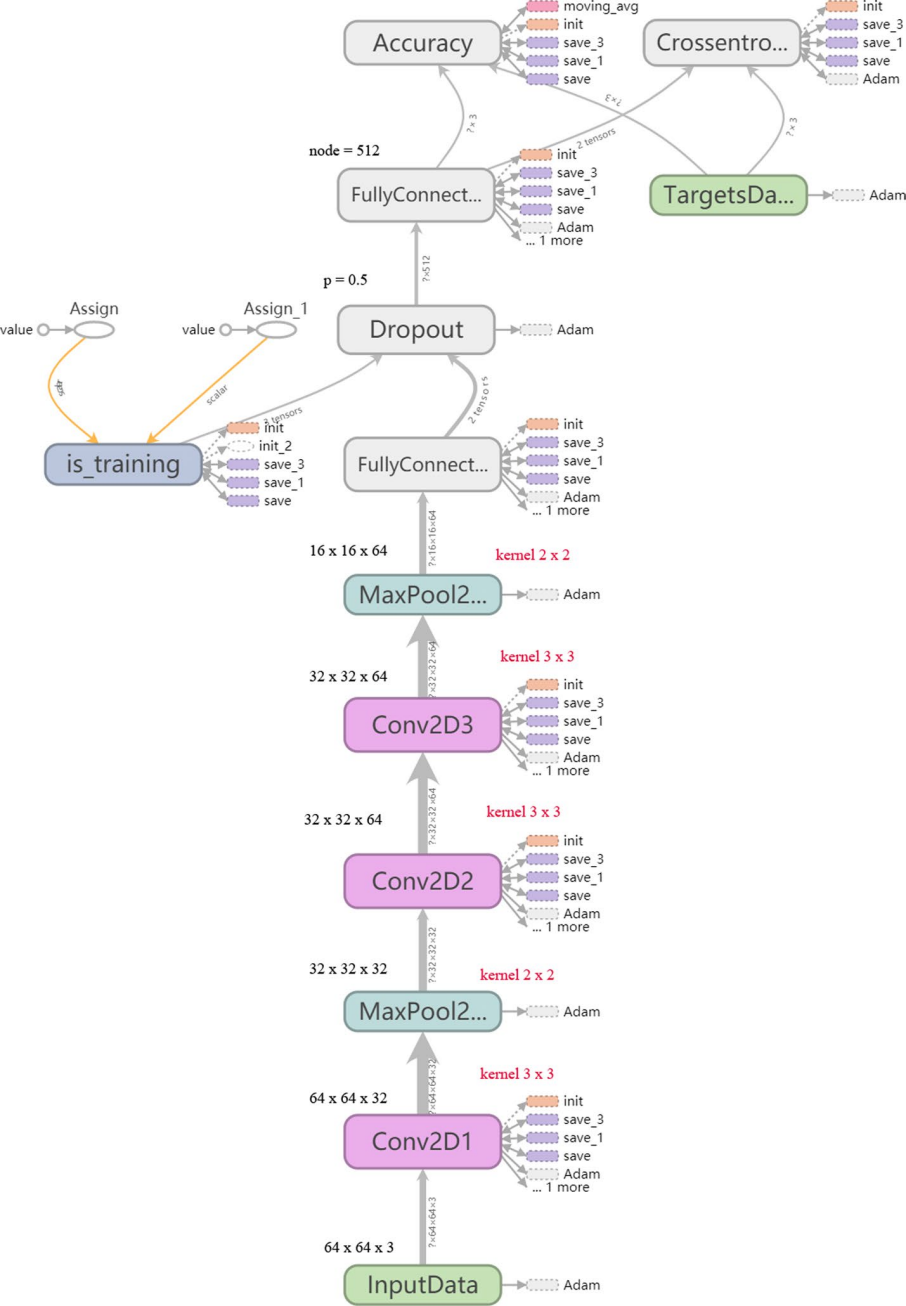
AlexNet-based CNN for fMRI data

We used a deep CNN that was based on AlexNet and limited by the amount of data: three convolutional layers, two pooling layers and two fully connected layers (Fig. 1). With respect to the input layer, the post-feature mapping fMRI images were used as input data, with the convolutional layer playing the most important role in the CNN architectures as it is the core building block of such networks. The parameters of the convolutional layer consist of a set of learnable filters, where very filter is spatially small but extends through the full depth of the input volume. In our study, the filters had a size of 3 × 3 and computed the output of the neurons that were connected to local regions in the input. Thus, each output is computed as a dot product between its weight and the region that it is connected to the input volume. The pooling layer performs a down-sampling operation along the spatial dimensions, and to reduce hyper parameters and control overfitting, we exploited the max pooling layer in our framework. The normalization layer also plays an important role in CNN architecture. Here we used the rectified linear units (ReLu) layer, which applies an element-wise activation function, such as max (0, x) thresholding at zero. This layer does not change the size of the image volume [28, 29]. Next, the fully connected (FC) layer, as the name implies, each neuron in this layer is connected to all the numbers in the previous volume [16], and computes the class scores, resulting in the volume of the number of classes. The main goal when using a dropout layer is to avoid overfitting [30], especially in cases of limited training data. The dropout layer randomly drops neurons with some probability, *p*, which in our study, is equal to 0.5. In addition, we also exploited Adam [31] as an optimizer, which is a simple and computationally efficient algorithm for gradient-based optimization of stochastic objective functions.

### Inception module

**Fig. 2:**
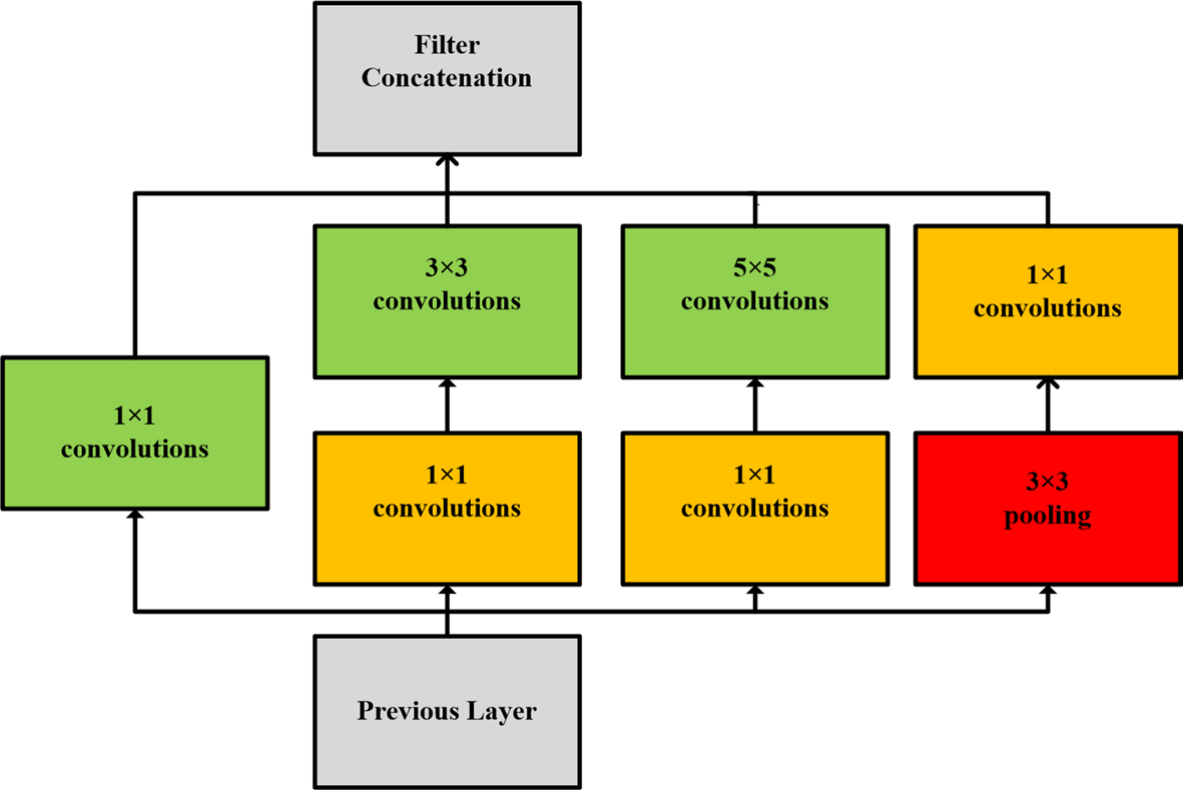
Inception module in our architecture

GoogleNet, which was developed by Szegedy et al. [18], is a successful network broadly used for object recognition and classification. The architecture of GoogleNet consists of a deep network based on a modern design module called Inception (Fig. 2). This module allows for a significant increase in the number of units at each layer without increasing computational complexity until later stages. This is achieved through a global dimensionally reduction prior to costly convolutions with larger patch sizes.

**Fig. 3:**
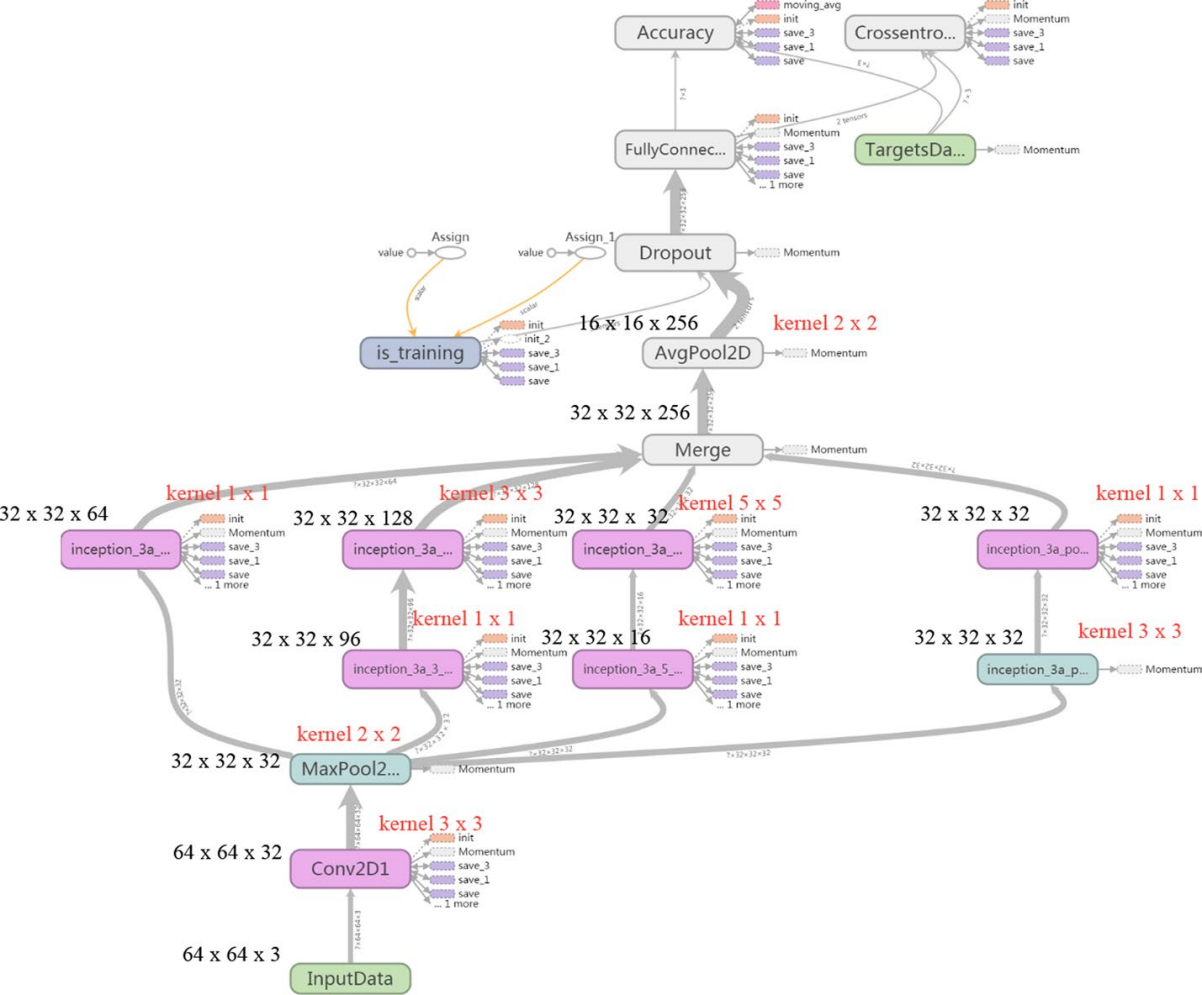
CNN with the Inception module for fMRI data

In our second frames, we use this module to improve the performance of the classifier. Before development of the Inception module, the fundamental approaches used to improve accuracy of CNN architecture were to increase the size of the layers and make the network deeper. However, this straightforward solution causes two major issues. First, a large number of hyper parameters require more training data and can result in overfitting, especially in the case of limited training data. Second, uniform increases in network size dramatically increase interactions with computational resources, thereby affecting both the timing performance and cost of providing infrastructure. In consideration of our datasets, our frame as shown in Fig. 3 (Additional file 1: TableS1).

### Training

**Fig. 4:**
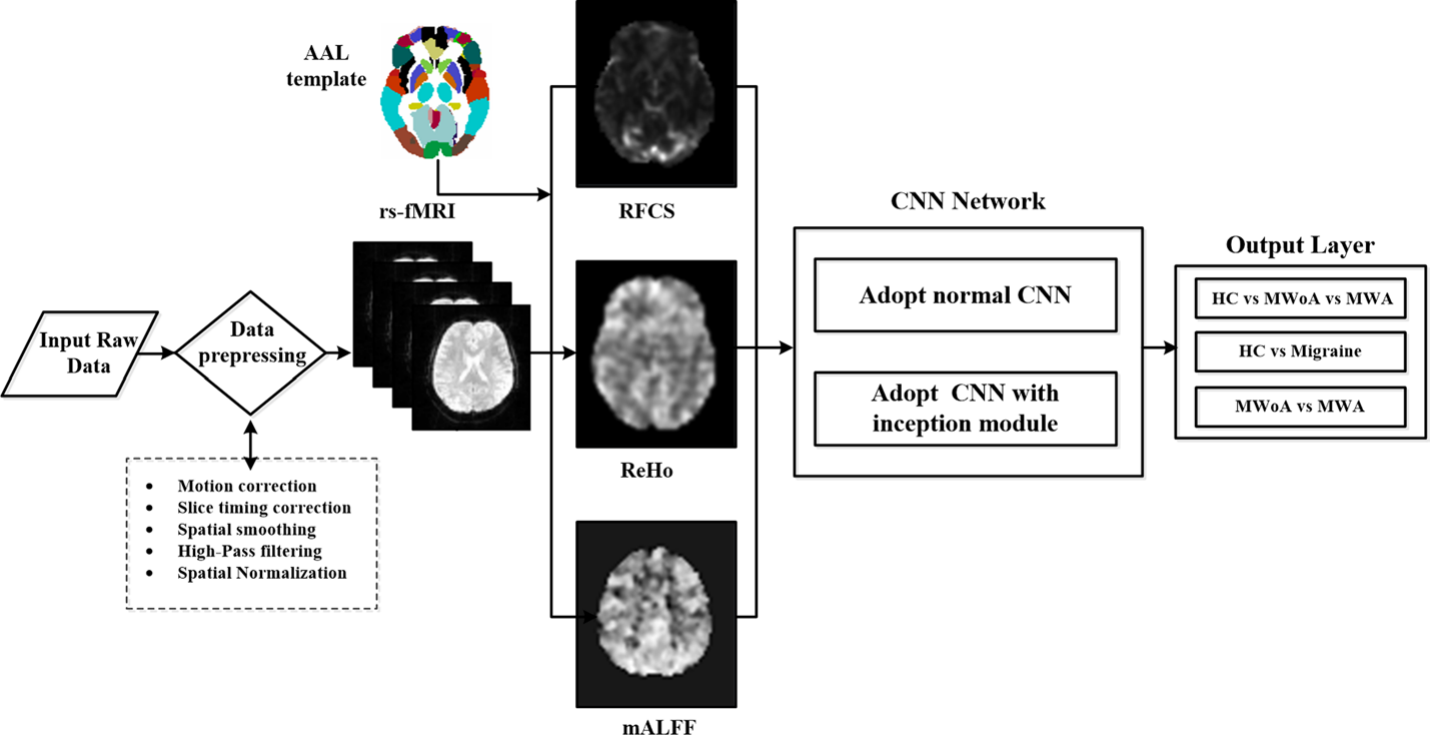
Schematic illustration of the classification. ALFF, ReHo, RFCS are used to map resting-state brain function, respectively. Two CNN networks are designed to classify a variety of groups

The preprocessed rs-fMRI time series data were first loaded into memory using the neuroimaging package Nibabel (http://nipy.org/nibabel/) and were then decomposed into 2D (x, y) matrices along z and time (t) axes. Next, the 2D matrices were converted to lossless PNG format using the Python and OpenCV (opencv.org). To improve the performance of the classifier, we removed some slices of each time course because they included no functional information. During the training process, about 80% of the subjects from the three groups (51 individuals in total) were assigned to the training dataset, and the remaining 20% were used for testing purposes. This ensures the relative independence between the training set and the testing set. In the data conversion process, a total of 5760 images were produced, including 1890 for MWoA, 1350 for MWA and 2520 for normal control PNG samples. These training and testing images were randomly shuffled and resized to 63 × 63 pixels. For these images, we have exploited data augmentation, such as cropping, rotating, and flipping input images [32], to avoid overfitting. Then they were converted to h5py-formatted data through Tensorflow, which is a Deep learning platform (http://www.tensorflow.org) used for this classification experiment. In our study, we divided the data into three groups, migraine vs. HC, HC vs. MWoA vs. MWA, and MWA vs. MWOA, and used them as three different inputs. Then, we adopted two different deep learning frameworks and compared the identification results obtained between the two. The general flow of the classification process is depicted in Fig. 4.

The weights of hidden layers are randomly initialized, and for the output layer, we choose the SoftMax function as the classifier. The numbers of the units in the output layer represent the conditional probabilities that the input belongs to each of the classes. In the training phase, the learning rate drops every 10 epochs; we started with 0.01 and divided it by 10 every 10 epochs. We used the cross-entropy as our cost function, *J (w, b)*, as it is commonly used for classification tasks [29]. This function is:

2 $$- \frac{1}{N}\sum\limits_{i = 1}^{N} {\sum\limits_{j = 1}^{J} {\left[ {{\rm I}\left\{ {\mathop {\text{y}}\nolimits^{i} = j} \right\}\log \left( {h_{w,b} \left( {x^{i} } \right)_{j} } \right)} \right]} }$$

where, *N* is the number of MRI images, *j* is the sum over the number of classes, *h*_*w,b*_ is the function computed by the network, and *x*, *y* are the input and label of image, respectively.

## Results

Here, we made classifications based on different feature types and used test examples (unseen examples) to evaluate the performance of the models. For robustness and reproducibility purposes, we repeated cross-validation of the deep neural networks four times (fourfold cross validation) [33]. The final accuracy of each CNN was subsequently computed by averaging the accuracies across the four runs (Tables 2, 3).

**Table 2:** The accuracy of testing dataset in CNN based on AlexNet is demonstrated

Feature	Data group	Accuracy
ALFF	HC vs. migraine	89.56% ± 0.24%
	HC vs. MWoA vs. MWA	87.31% ± 0.26%
	MWoA vs. MWA	86.43% ± 0.54%
ReHo	HC vs. migraine	93.42% ± 0.13%
	HC vs. MWoA vs. MWA	92.66% ± 0.21%
	MWoA vs. MWA	87.39% ± 0.27%
RFCS	HC vs. migraine	98.63% ± 0.28%
	HC vs. MWoA vs. MWA	98.78% ± 0.36%
	MWoA vs. MWA	96.87% ± 0.43%

**Table 3:** The accuracy of testing dataset in CNN with Inception module is demonstrated below

Feature	Data group	Accuracy
ALFF	HC vs. migraine	92.38% ± 0.14%
	HC vs. MWoA vs. MWA	92.31% ± 0.15%
	MWoA vs. MWA	89.77% ± 0.39%
ReHo	HC vs. migraine	95.07% ± 0.19%
	HC vs. MWoA vs. MWA	94.81% ± 0.35%
	MWoA vs. MWA	93.44% ± 0.20%
RFCS	HC vs. migraine	99.25% ± 0.36%
	HC vs. MWoA vs. MWA	98.69% ± 0.29%
	MWoA vs. MWA	96.13% ± 0.22%

The best performer out of three different features was the RFCS, and the highest identification rate achieved was 99.25% when using the Inception module-based CNN to distinguish between the HC and migraine groups. According to the experimental data, the recognition rate of the AlexNet-based CNN was lower than that of the Inception module-based CNN, especially in terms of the ALFF feature. As expected, the Inception module-based CNN improved the classification performance in most cases. It was relatively hard for either framework to distinguish between the MWoA and MWA groups, with the AlexNet-based CNN showing an identification rate of just 86.43%. In addition, compared with other features, the RFCS feature mapping improved the classification accuracy of both of the deep learning-based models, with a noticeable difference between the two.

### ROC curve

**Fig. 5:**
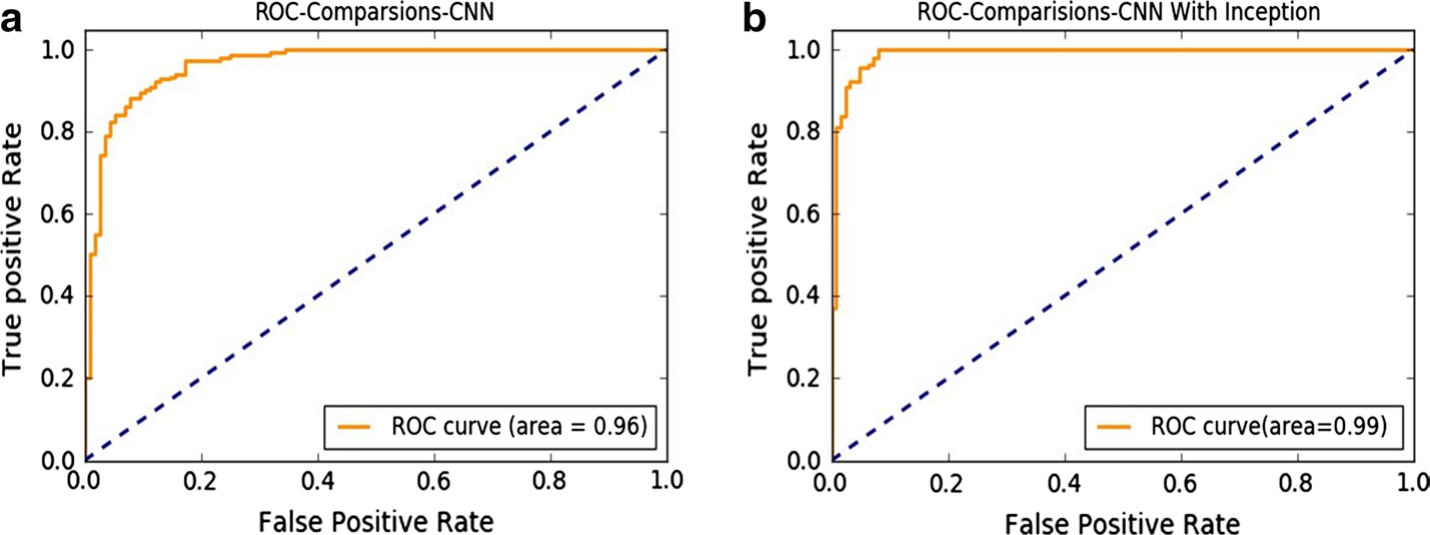
ROC curves show the tradeoff between sensitivity (y-axis) and specificity (x-axis) for the **a** AlexNet-based CNN and **b** Inception module-based CNN

The area under the receiver operating characteristic (ROC) curve is the most popular metric to measure the performance of a classifier [34]. It is a plot of the true positive rate against the false positive rate. Here, we calculated the true positive, true negative, false positive, and false negative rates from 0 to 1 based on the prediction results. Next, for each classifier, the sensitivity, specificity and accuracy were calculated as follows:

3 $$Sensitivity = True\;Positive\;Rate\;(TPR) = \frac{TP}{P} = \frac{TP}{TP + FN}$$

4 $$Specificity = True\;Negative\;Rate\;(TNR) = \frac{TN}{N} = \frac{TN}{FP + TN}$$

5 $$Accuracy = \frac{TP + TN}{TP + FN + FP + TN}$$

where, in the case of the HC vs. migraine group, TP denotes the number of patients correctly classified, FN denotes the number of controls correctly predicted, and FP denotes the number of controls classified as patients. To evaluate the performance of the two classifiers, we plotted the ROC (Fig. 5) and calculated the area under curve (AUC), for which a greater AUC represents a more accurate test (better classification). For comparison purposes, we focused on the RFCS feature and the HC vs. migraine group. We found an AUC of 0.99 for the Inception-based CNN, a value that indicates an excellent discrimination power.

## Discussion

Here, we examined the ability of deep learning-based frameworks to discriminate between MWoA, MWA, and HC using features extracted from rs-fMRI data. Our results indicate that with each of these features—ALFF, ReHo and RFCS—it is possible to achieve relatively high accuracies when classifying the three groups of datasets. Noteworthy, in both CNN models, highest accuracies were achieved when using the RFCS feature. Upon comparing the results obtained using the two different CNNS, we found that the Inception module-based CNN shows a better performance than the AlexNet-based CNN. Compared with the support vector machine classifier that we previously analyzed (a final classification accuracy of 83.67%) [20], our approach provides preliminary support for deep learning methods combined with the fMRI features as a method for improving the discriminative power for migraine. In fact, we obtained an accuracy as high as 99.25% when using the RFCS feature in deep learning-based frameworks. Resting-state functional connectivity has been defined as the identification of correlation patterns between fluctuations measured in different brain areas [35]. The RFCS measures the average correlation of a given brain region with all other regions. Our results suggest that RFCS holds the potential to increase the translation of fMRI data into clinical diagnosis.

Many studies have reported the existence of strong functional connections in the resting state [36]. These resting-state networks consist of anatomically separated, but functionally linked brain regions that show a high level of ongoing functional connectivity during rest. For example, abnormal functional connectivity has been reported in the prefrontal and temporal regions [11] and in the amygdala and visceroceptive cortex [10] of migraine sufferers (as compared to healthy control individuals). These regions may thereby contribute more in the classification. When measuring the functional connectivity among regions, global alterations emerge that may carry important information for enhancing accuracy rates. This could explain the higher levels of accuracy that we found in the present study. In addition, we found that considering ReHo and ALFF also led to good performance levels. Thus, we conclude that the deep learning-based frameworks can help identify migraine patients when using these fMRI features. We also noticed that classification accuracies were lower when comparing MWoA with MWA, especially in the ALFF group. Perhaps all subjects in the MWoA vs. MWA group experienced headache symptoms and as such, had more similar imaging markers as compared with the HC group.

For all features tested, the Inception module-based CNN resulted in a higher level of accuracy than the AlexNet-based CNN. A considerable amount of research has demonstrated the feasibility and effectiveness of the Inception module by stacking the convolution layers and pooling layers of different scales, which allows for training a high-quality network on training sets of relatively modest sizes. Szegedy et al. [18] exploited Inception models, reaching a multi-crop evaluation top-5 error of 3.5% (a 25% reduction with respect to the best published value). In our models, the Inception module uses parallel 1 × 1, 3 × 3, and 5 × 5 convolutions along with a max-pooling layer in parallel. These characteristics enable our models to capture a variety of features in the parallel and have a filter concatenation layer. This type of structure helps to automatically choose the appropriate kernel size in convolutional layers, as well as to concatenate the outputs of all these parallel layers. This in turn contributes to the combination of different sized features and it is helpful in the classification of migraine. Our approach provides initial evidence for a rapid and accessible method that has the potential to aid in making clinical decisions.

Besides identifying migraine and HC, our method was also able to distinguish between MWoA and MWA data. These two subtypes of migraine likely reflect differences in pathogenesis, and thus, the ability to identify different subtypes of migraine presents an advantage for clinical diagnoses and treatment. Using fMRI, Datta et al. [37] compared the responsiveness of MWA, MWoA and HC to visual stimuli and found that only the occipital and lateral geniculate cortex of MWA patients exhibited high reactivity. This high reactivity in the visual cortex is believed to be directly related to the aura. As the subtypes of migraine differ in their symptoms, choosing the most appropriate treatments for each subtype is a major challenge. Our research makes it possible to identify subtypes of migraine headache based on differences in precipitating factors, an achievement that will prove useful in clinical diagnosis.

Several limitations of this study should be noted. First, we only included 5760 images in the deep learning-based frameworks, the dataset was indeed small. To avoid overfitting, the frameworks we exploited were relatively shallow. In neuroimaging studies using data from migraine patients, it is difficult to obtain a large number of samples. However, the recent trend in the research community toward a greater level of sharing of neuroimaging data should increase the availability of training sets for future studies. The use of larger datasets would be useful to determine whether or not our approach can be applied on a larger scope and be used to make generalizations. Second, although we tried to extract the features, ALFF, ReHo and RFCS features to improve the performance of the classifier, there is no combination of models that relate to specific brain regions. This in fact, should be the aim of further research. Moreover, there is still no definite conclusion as to whether using the original rs-fMRI or the image after feature mapping in the deep learning framework generates better results. Nonetheless, our group has achieved good performances in the discrimination of post-traumatic stress disorder (PTSD) using these three indices [38]. Third, for this method to be applied in clinical practice, it is important to enhance transparency (due to the “black box” models) and generate a higher level of trust. Based on our results of these deep learning-based frameworks, our future goal is to visualize the brain regions that are most affected by migraines. Altogether, this information will ultimately help strengthen our understanding of the pathogenesis of this disorder.

## Conclusion

Compared with traditional support vector machine, we were able to improve classification performance using CNN. Moreover, RFCS, ReHo and ALFF, these three functional indices we employed, can be used to represent different degrees of classification features. RFCS, which reflects the global alterations for one subject, carries important information for enhancing accuracy rates and obtaining the optimum classification results. Furthermore, using these three functional measures of rs-fMRI combined with deep learning frameworks is a powerful method that can potentially improve the clinical diagnosis of migraine and other brain disorders.

## Additional file

**Additional file 1: Table S1.** The architecture of convolutional embedding function.

## Abbreviations

CNN: convolutional neural network
MWoA: migraine without aura
MWA: migraine with aura
fMRI: resting-state functional magnetic resonance imaging
ALFF: amplitude of low-frequency fluctuations
ReHo: regional homogeneity
RFCS: regional functional correlation strength
BOLD: blood oxygen level-dependent
ROC: receiver operating characteristic
