# Supplementary material for: Multimodal MRI-based classification of migraine: using deep learning convolutional neural network
Source: Biomed Eng Online. 2018 Oct 11;17:138. doi: 10.1186/s12938-018-0587-0 (PMC6186044; doi:10.1186/s12938-018-0587-0)
Supplement: Supplementary file 1 — Additional file 1: Table S1. The architecture of convolutional embedding function. [file 12938_2018_587_MOESM1_ESM.docx]

| Layer | filters | size | stride | padding | Output |
| --- | --- | --- | --- | --- | --- |
| Convolutional | 32 | 3 x 3 | 1 | 1 | 32 x 64 x 64 |
| Maxpool |  | 2 x 2 | 2 | / | 32 x 32 x 32 |
| Convolutional | 64 | 3 x 3 | 1 | 1 | 64 x 32 x 32 |
| Convolutional | 64 | 3 x 3 | 1 | 1 | 64 x 32 x 32 |
| Maxpool |  | 2 x 2 | 2 | / | 64 x 16 x 16 |
| FC |  |  |  |  | 1 x 1 x 512 |
| SoftMax |  |  |  |  | / |

| Layer | filters | size | stride | padding | Output |
| --- | --- | --- | --- | --- | --- |
| Convolutional | 32 | 3 x3 | 1 | 1 | 32 x 64 x 64 |
| Maxpool |  | 2 x 2 | 2 |  | 32 x 32 x 32 |
| Inception3a_conv | 64 | 1 x 1 | 1 |  | 64 x 32 x 32 |
| Inception3a_conv | 96 | 1 x 1 | 1 |  | 96 x 32 x 32 |
| Inception3a_conv | 128 | 3 x 3 | 1 | 1 | 128 x 32 x 32 |
| Inception3a_conv | 16 | 1 x 1 | 1 |  | 16 x 32 x 32 |
| Inception3a_conv | 32 | 5 x 5 | 1 | 2 | 32 x 32 x 32 |
| Inception3a_pool |  | 3 x 3 | 1 | 1 | 32 x 32 x 32 |
| Inception3a_conv | 32 | 1 x 1 | 1 |  | 32 x 32 x 32 |
| Merge_inception | / | / | / |  | 256 x 32 x 32 |
| AvgPool |  | 2 x 2 | 1 |  | 256 x 16 x 16 |
| SoftMax |  |  |  |  | / |

Table S1. The architecture of convolutional embedding function
